# Exploring the bidirectional relationship between metabolic syndrome and thyroid autoimmunity: a Mendelian randomization study

**DOI:** 10.3389/fendo.2024.1325417

**Published:** 2024-03-19

**Authors:** Kefan Chen, Wei Sun, Liang He, Wenwu Dong, Dalin Zhang, Ting Zhang, Hao Zhang

**Affiliations:** Department of Thyroid Surgery, The First Hospital of China Medical University, Shenyang, Liaoning, China

**Keywords:** metabolic syndrome, thyroid autoimmunity, TPOAb-positivity, Mendelian randomization, GWAS

## Abstract

**Background:**

Observational studies have reported a possible association between metabolic syndrome (MetS) and thyroid autoimmunity. Nevertheless, the relationship between thyroid autoimmunity and MetS remains unclear. The objective of this research was to assess the causal impact of MetS on thyroid autoimmunity through the utilization of Mendelian randomization (MR) methodology.

**Methods:**

We performed bidirectional MR to elucidate the causal relationship between MetS and their components and thyroid autoimmunity (positivity of TPOAb). Single nucleotide polymorphisms (SNPs) of MetS and its components were obtained from the publicly available genetic variation summary database. The Thyroidomics Consortium conducted a genome-wide association analysis, which provided summary-level data pertaining to thyroid autoimmunity. The study included several statistical methods, including the inverse variance weighting method (IVW), weighted median, simple mode, weight mode, and MR-Egger methods, to assess the causal link. In addition, to ensure the stability of the results, a sensitivity analysis was conducted.

**Results:**

IVW showed that MetS reduced the risk of developing thyroid autoimmunity (OR = 0.717, 95% CI = 0.584 - 0.88, P = 1.48E−03). The investigation into the causative association between components of MetS and thyroid autoimmune revealed a statistically significant link between triglycerides levels and the presence of thyroid autoimmunity (IVW analysis, OR = 0.603, 95%CI = 0.45 -0.807, P = 6.82E−04). The reverse analysis did not reveal any causal relationship between thyroid autoimmunity and MetS, including its five components.

**Conclusions:**

We have presented new genetic evidence demonstrating that MetS and its triglyceride components may serve as potential protective factors against thyroid autoimmunity.

## 1 Introduction

Autoimmune thyroid disease (AITD), encompassing Hashimoto’s thyroiditis (HT) and Graves’ disease (GD), represents a prevalent autoimmune disorder, with a prevalence rate ranging from 2% to 5% among the general population in western countries (1, 2). Anti-thyroid peroxidase antibodies (TPOAb) are frequently detected in HT patients, this enzyme is located in the thyroid gland and plays a key role in thyroid hormone synthesis, predicting the eventual development of HT (3, 4). The prevalence of thyroid autoimmune illness is notably high among women in their reproductive years, with the most prevalent manifestation being TPOAb-positivity. This condition is associated with an elevated likelihood of experiencing pregnancy difficulties, including miscarriage and preterm birth (5–7). In addition, thyroid autoimmunity may be an independent risk factor for cardiovascular disease by promoting chronic inflammation (8, 9). TPOAb serve as a valuable clinical indicator in the identification of early AITD. The prevalence of AITD has been escalating, rendering it a significant public health concern in contemporary times (10).

Metabolic syndrome (MetS), a widely recognized risk factor for cardiovascular disease, comprises five components: hypertension, elevated blood glucose (HBG) and triglycerides levels, low high-density lipoprotein cholesterol(HDL-C), and increased waist circumference (WC) (11, 12). When a person has three or more of the components described above, they can be diagnosed with MetS. A recent meta-analysis conducted on a large-scale dataset comprising 28 million persons worldwide has provided an estimation of the global prevalence of MetS. The research revealed that the prevalence of MetS falls between the range of 12.5% to 31.4%, according to the different definitions used (13). MetS are estimated to affect more than 1 billion people worldwide (14). MetS and its components have been linked to various diseases and increased death rates, hence posing a significant health concern and imposing a substantial economic burden (15, 16).

Limited knowledge known regarding the correlation between thyroid autoimmunity and MetS. A research conducted in Korea revealed that individuals diagnosed with MetS exhibited a significantly elevated rate of TPOAb-positivity (17). This finding implies a potential association between thyroid autoimmunity and MetS. Nevertheless, a separate study indicated that TPOAb-positivity did not exert any influence on the occurrence of MetS among postmenopausal women who exhibited normal thyroid function (18). Another study from Portugal found that TPOAb- positivity was negatively associated with MetS (19). So we can see that there may be a potential causal relationship between TPOAb-positivity and MetS. However, it’s important to recognize that associations between TPOAb-positivity, MetS, and its components in observational studies may be influenced by confounding factors, small sample sizes, limited follow-up, and reverse causation, potentially leading to misinterpretations (20). Hence, the causative relationship between TPOAb-positivity and the risk of MetS and its components remains uncertain, and the reverse scenario is equally uncertain.

Mendelian randomization (MR), which uses single nucleotide polymorphism (SNP) as a proxy for lifetime exposure risk, can reduce confounding factors and reverse causation bias and can overcome the limitations of observational studies (21, 22). This method is advantageous for investigating causality since genetic variation is randomly allocated during meiosis, thereby reducing the impact of confounding variables, measurement inaccuracies, and the issue of reverse causality that can affect conventional multivariate regression techniques (23). In this research, we employed a bidirectional two-sample MR approach to investigate the reciprocal causation between TPOAb-positivity and MetS and its components. The objective is to contribute insights for the development of more efficacious interventions for these conditions.

## 2 Materials and methods

### 2.1 Study design

**Figure 1:**
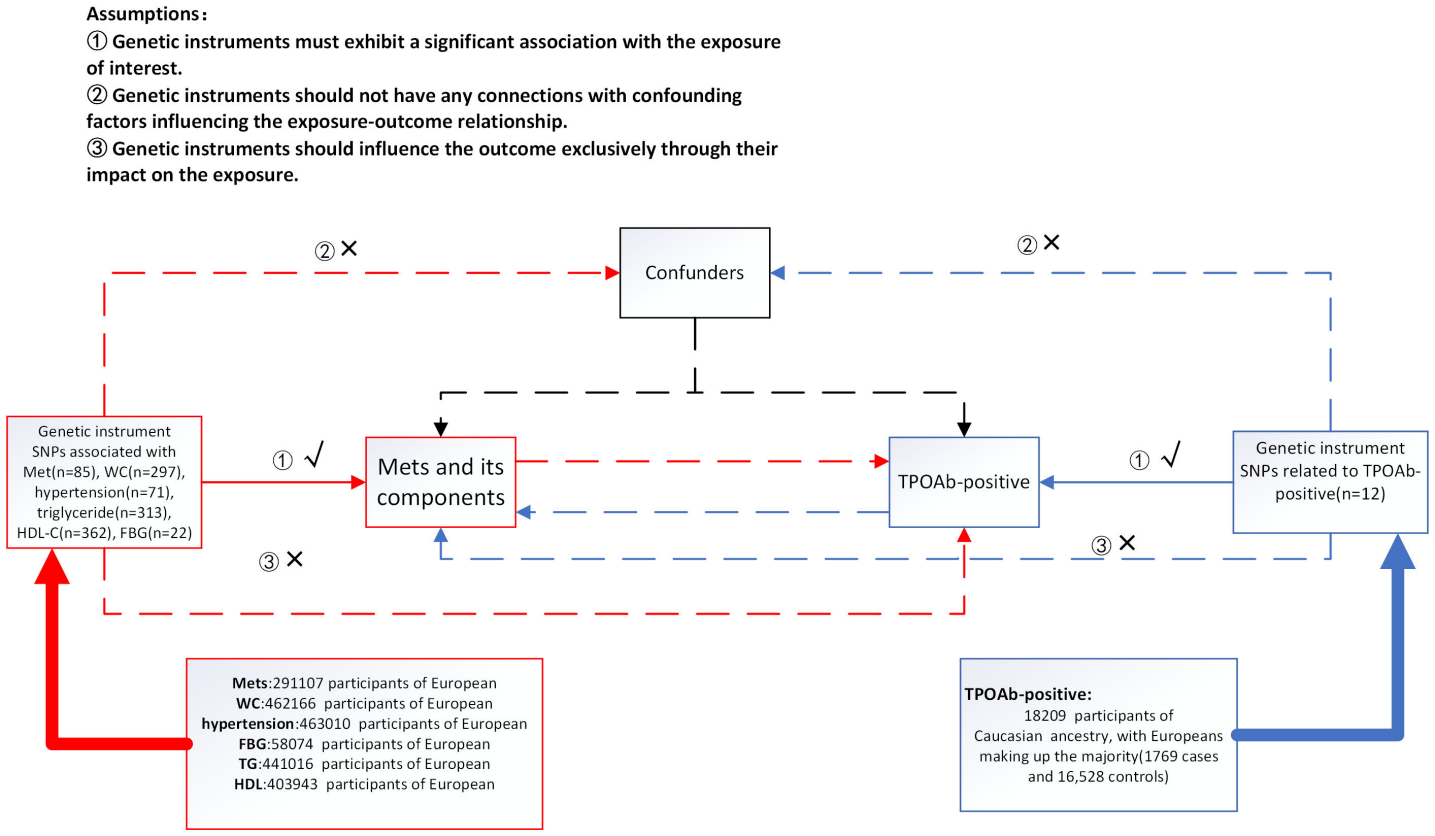
Summary of the research design in this bidirectional Mendelian randomization (MR) study. We performed a total of 12 MR analyses to investigate the bidirectional association between MetS and its components and TPOAb-positivity. MetS metabolic syndrome, FBG fasting blood glucose, WC waist circumference, HDL-C high-density lipoprotein cholesterol.

In our current MR investigation, we utilize a bidirectional framework, with instrumental variables (IVs) derived from seven genome-wide association studies(GWAS), to assess both the IV-exposure and IV-outcome associations. For MetS, we used the National Cholesterol Education Program Adult Treatment Panel III (NCEP/ATP III) diagnostic criteria, which contain five diagnostic elements (24). Initially, we acquired data pertaining to the exposure variables, including Metabolic Syndrome (MetS), hypertension, waist circumference, fasting blood glucose (FBG), serum triglycerides, and HDL-C, from the GWAS database to identify SNPs associated with these exposure factors. Subsequently, we employed an other GWAS database to access the data related to the outcome variables, namely, TPOAb-positivity. According to the description of the GWAS study, it used TPOAb-positivity thresholds provided by different assay manufacturers, rather than using a fixed threshold, but the results were not heterogeneity and were reliable (25). Ultimately, eligible SNPs were chosen, and MR analysis was employed to ascertain the causal relationship between the exposure factors and the risk of the outcome variable. The roles of exposure factors and outcome variables were interchanged in each analysis to explore the potential presence of reverse causality between the two. MR relies on three fundamental assumptions(**Figure 1**): ① Genetic instruments must exhibit a significant association with the exposure of interest. ② Genetic instruments should not have any connections with confounding factors influencing the exposure-outcome relationship. ③ Genetic instruments should influence the outcome exclusively through their impact on the exposure (26). In order to prevent the influence of different ethnic factors on the results, only the European population was included in the study.

### 2.2 Selection of IVs for MR analyses

For each exposure factor, SNPs were screened based on adherence to the three primary assumptions of MR. The IVs are established based on the following criteria (1) SNPs exhibiting genome-wide significance (P < 5 × 10^-8^). As TPOAb-positive SNPs are screened too strictly according to this criterion, when using TPOAb-positive SNPs as exposure factors, a threshold of ‘P < 5 × 10^-6^’ was applied for filtering. (2) To eliminate linkage disequilibrium among these SNPs, we utilized the 1,000 Genomes European panel as the reference population, with an r² threshold of 0.001 and clumping distance of 10,000 kb. (3) Harmonization procedures were carried out to eliminate ambiguous and palindromic SNPs. (4) The RadialMR method was used to identify and remove outliers, which ultimately reduced heterogeneity and horizontal pleiotropy and made the results more reliable (27). (5) In order to gauge the strength of the instrumental variables, we computed F-statistics. Typically, an F-statistics threshold exceeding 10 is recommended for MR analyses (28).

### 2.3 Data sources and IVs selection for MetS and their component

Summary-level data for MetS were sourced from the extensive GWAS conducted within the UK Biobank (**Table 1**), encompassing a dataset of 291,107 individuals of European descent, consisting of 59,677 cases and 231,430 controls, all with complete data on genotypes, outcomes, and covariates (29). In total, 85 independent genetic SNPs meeting genome-wide significance criteria (p < 5 × 10^-8^) were identified and chosen as genetic instruments for MetS.

**Table 1:** TPOAb-positive, Mets and its components GWAS datasets.

	TPOAb-positive	Mets	WC	hypertension	FBG	Triglycerides	HDL-C
Year	2014	2019	2018	2018	2012	2020	2020
Population	Europeans	Europeans	Europeans	Europeans	Europeans	Europeans	Europeans
Sex	male and female	male and female	male and female	male and female	male and female	male and female	male and female
Ncase	1,769	59,677	NA	54358	NA	NA	NA
Ncontrol	16,528	231,430	NA	408652	NA	NA	NA
Sample size	18,209	291,107	462,166	463,010	58,074	441,016	403,943
Data sources	Thyroidomics Consortium	UK Biobank	MRC-IEU	MRC-IEU	Alisa K Manning et al.	UK Biobank	UK Biobank
GWAS ID	NA	NA	ukb-b-9405	ukb-b-12493	ebi-a-GCST005186	ieu-b-111	ieu-b-109

Mets, Metabolic syndrome. WC, waist circumference. FBG, fasting blood glucose. TG, triglycerides. HDL-C, high-density lipoprotein cholesterol. GWAS, genome-wide association studies. NA, not available.

Genetic instruments for WC were obtained from the Medical Research Council Integrative Epidemiology Unit (MRC-IEU) UK Biobank GWAS pipeline, comprising a dataset of 462,166 individuals of European descent. When WC was used as an exposure factor, 297 SNPs were extracted as instrumental variables (p < 5 × 10−8).

As for hypertension, data was similarly sourced from the MRC-IEU UK Biobank pipeline. The dataset encompassed 54,358 cases and 408,652 controls, all of European ancestry. After screening (p < 5 × 10^-8^), a total of 71 SNPs were integrated into the analysis.

Regarding HDL-C and triglycerides, summary statistics were acquired from the UK Biobank, comprising a dataset of over 400,000 individuals of European descent (30). Following screening, a total of 362 SNPs for HDL-C and 313 SNPs for triglycerides were ultimately incorporated into the analysis, with a significance threshold of p < 5 × 10^-8^.

Genetic instruments for FBG were sourced from a database containing 58,074 individuals of European descent (31). The dataset was appropriately adjusted for body mass index (BMI). After the screening process, a total of 22 SNPs were recognized for their robust association with FBG and were subsequently integrated into the study, adhering to a significance threshold of p < 5 × 10^-8^.

### 2.4 GWAS summary-level data for TPOAb-positivity

Genetic variation data for TPOAb-positivity was obtained from the Thyroidomics Consortium (**Table 1**). The summary-level statistics originate from a substantial study encompassing 1,769 cases and 16,528 controls, all of Caucasian ancestry, with a predominant representation of individuals of European descent (25). Subsequent to the screening process, a total of 12 SNPs demonstrated robust associations with TPOAb-positivity and were thus incorporated into the analysis, adhering to a significance threshold of p < 5 × 10^-6^.

### 2.5 Statistical analyses

In our analysis, we employed the inverse-variance weighted (IVW) MR method to estimate the relationships between MetS and its components and TPOAb-positivity. This method offers a reliable causal estimate, particularly in the absence of directional pleiotropy. Additionally, we conducted alternative analyses using the weighted median, simple mode, weight mode, and MR-Egger methods. Sensitivity analysis methods encompassed Cochran’s Q test, funnel plot analysis, leave-one-out (LOO) analyses, and MR-Egger intercept tests. Heterogeneity among genetic instruments was assessed using Cochran’s Q test (32). Additionally, the MR-Egger intercept was utilized to examine potential horizontal pleiotropy through the intercept term (33). LOO tests were employed to investigate whether the inference of a causal association is influenced by a single SNP. This was achieved by conducting repeated IVW analyses while sequentially excluding each SNP associated with the exposure.

Statistical significance is set at the threshold of the Bonferroni-corrected P< 0.004 (0.05/12) in this study. F-statistics were computed to gauge the robustness of the relationship between instrumental variables and exposure factors (34). All MR analyses were conducted utilizing the TwoSampleMR and RadialMR R packages within R software version 4.2.1.

## 3 Results

### 3.1 The causal impact of MetS and its components on TPOAb-positivity

**Figure 2:**
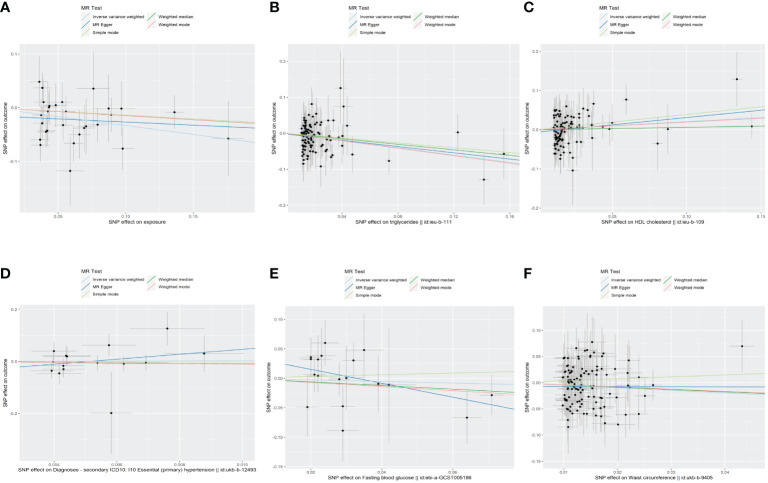
The scatter plots of the association between genetic predicted MetS and its components on TPOAb-positivity in MR analysis. **(A)**MetS on TPOAb-positivity; **(B)** triglycerides on TPOAb-positivity; **(C)** HDL-C on TPOAb-positivity; **(D)** hypertension on TPOAb-positivity; **(E)** FBG on TPOAb-positivity; **(F)** WC on TPOAb-positivity. MetS, metabolic syndrome; HDL-C, high-density lipoprotein cholesterol; FBG, fasting blood glucose; WC, waist circumference.

In the MR analysis, following the exclusion of SNPs that were not available in the summary-level dataset for MetS, as well as the removal of palindromic SNPs, and the elimination of abnormal SNPs post RadialMR screening, we employed 29 genetic variants as instruments for MetS, 114 variants for WC, 15 variants for hypertension, 98 variants for serum triglycerides, and 100 variants for HDL-C. Additionally, 18 variants were utilized as instruments for FBG. The results of the MR analysis and sensitivity analysis are outlined in **Table 2**, and scatter plots are provided in **Figures 2**. Furthermore, it’s noteworthy that all SNPs exhibited F-statistics exceeding 10, which enabled the MR analysis (**Supplementary Table 1**).

**Table 2:** Genetic predicted MetS and its components on risk of TPOAb-positivity in the MR analysis.

Exposure	Outcome	Method	Nsnp	Pval	OR (95% CI)	Cochran Q test P-value	P-Egger_intercept
Metabolic syndrome	TPOAb-positivity	MR Egger	29	0.64	0.890(0.550-1.441)	0.666	0.34
		Weighted median		0.311	0.860(0.642-1.152)		
		IVW		1.48E-03	0.717(0.584-0.880)	0.666	
		Simple mode		0.575	0.869(0.536-1.410)		
		Weighted mode		0.402	0.861(0.609-1.216)		
FBG	TPOAb-positivity	MR Egger	18	0.076	0.308(0.091-1.039)	0.7	0.081
		Weighted median		0.424	0.735(0.345-1.565)		
		IVW		0.615	0.874(0.517-1.478)	0.517	
		Simple mode		0.833	1.159(0.301-4.461)		
		Weighted mode		0.412	0.704(0.311-1.594)		
WC	TPOAb-positivity	MR Egger	114	0.974	0.974(0.201-4.720)	0.648	0.55
		Weighted median		0.224	0.644(0.317-1.309)		
		IVW		0.032	0.613(0.392-0.959)	0.664	
		Simple mode		0.694	1.444(0.232-8.974)		
		Weighted mode		0.613	0.662(0.134-3.268)		
Hypertension	TPOAb-positivity	MR Egger	15	0.255	1.350E+04(0.002-8.44E+10)	0.508	0.255
		Weighted median		0.706	0.399(0.003-47.158)		
		IVW		0.891	1.283(0.036-46.115)	0.476	
		Simple mode		0.98	0.907(0.001-1.50E+3)		
		Weighted mode		0.726	0.364(0.001-93.534)		
Triglycerides	TPOAb-positivity	MR Egger	98	0.078	0.648(0.402-1.044)	0.964	0.711
		Weighted median		0.11	0.680(0.424-1.092)		
		IVW		6.82E-04	0.603(0.450-0.807)	0.969	
		Simple mode		0.495	0.716(0.275-1.865)		
		Weighted mode		0.02	0.594(0.386-0.916)		
HDL-C	TPOAb-positivity	MR Egger	100	0.06	1.451(0.988-2.13)	0.965	0.261
		Weighted median		0.758	1.063(0.721-1.567)		
		IVW		0.114	1.232(0.951-1.595)	0.962	
		Simple mode		0.359	1.478(0.644-3.389)		
		Weighted mode		0.308	1.216(0.837-1.767)		

IVW inverse-variance weighted, MetS metabolic syndrome, FBG fasting blood glucose, HDL-C high-density lipoprotein cholesterol.

**Figure 3:**
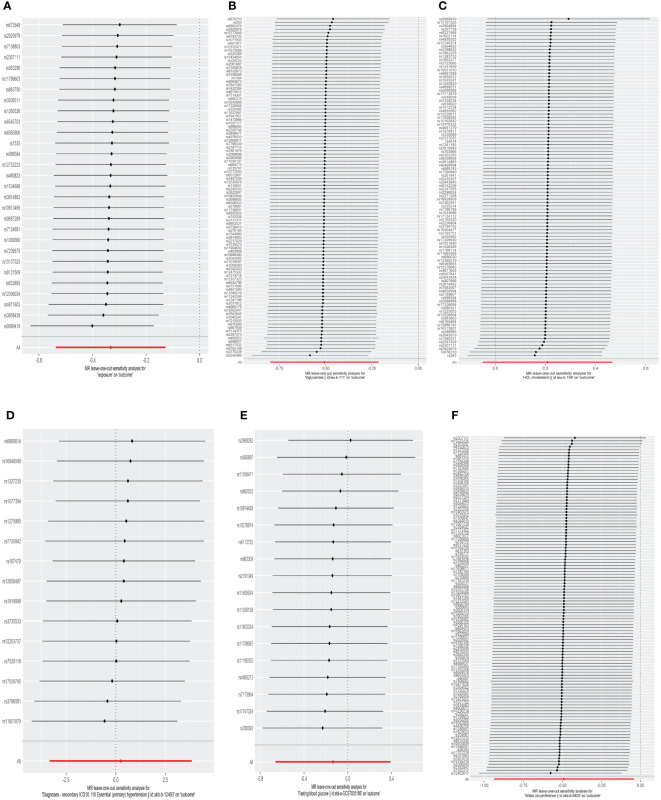
The leave-one-out analysis of the association between genetic predicted MetS and its components on TPOAb-positivity in MR analysis. **(A)**MetS on TPOAb-positivity; **(B)** triglycerides on TPOAb-positivity; **(C)** HDL-C on TPOAb-positivity; **(D)** hypertension on TPOAb-positivity; **(E)** FBG on TPOAb-positivity; **(F)** WC on TPOAb-positivity. MetS, metabolic syndrome; HDL-C, high-density lipoprotein cholesterol; FBG, fasting blood glucose; WC, waist circumference.

The results of the MR analyses were shown in **Table 2**, the scatter plots were presented in **Figure 2**. Our study reveals a negative causal association between MetS and TPOAb-positivity. The IVW analysis results (OR = 0.717, 95% CI = 0.584–0.880, P = 0.001) indicate that MetS serves as a protective factor against TPOAb-positivity. While not statistically significant, the MR-Egger results (OR = 0.890, 95% CI = 0.550–1.441), Weighted median (OR = 0.860, 95% CI = 0.642–1.152), Simple mode (OR = 0.869, 95% CI = 0.536–1.410), and Weighted mode (OR = 0.861, 95% CI = 0.609–1.216) continue to suggest that MetS reduces the risk of TPOAb-positivity. The MR-Egger (P = 0.666) and IVW (P= 0.666) P-values from the Cochran Q-test demonstrated the absence of heterogeneity in our findings, and no significant MR-Egger intercept values were detected (P = 0.340). The funnel plots exhibited symmetry (**Supplementary Figure S1**), and during the LOO sensitivity analysis, no single SNP was found to exert a significant impact on the overall outcomes (**Figure 3**). These observations collectively reinforce the robustness and stability of the results obtained from our MR analysis.

Concerning its components, the genetic predisposition to elevated triglycerides was found to be inversely associated with TPOAb-positivity. Specifically, the IVW method produced statistically significant results (OR = 0.603, 95% CI = 0.450–0.807, P = 6.816E^-04^). The other four methods, although lacking statistical significance, still hinted at a negative correlation between triglycerides and TPOAb-positivity. Specifically, the MR-Egger method (OR = 0.648, 95% CI = 0.402 - 1.044, P = 0.078), the Weighted median method (OR = 0.680, 95% CI = 0.424 - 1.092, P = 0.11), the Simple mode method (OR = 0.716, 95% CI = 0.275 - 1.865, P = 0.495), and the Weighted mode method (OR = 0.594, 95% CI = 0.386-0.916, P = 0.02) provided insights into this potential relationship. Furthermore, both the Cochran Q test (IVW, P = 0.969; MR-Egger, P = 0.964) indicated the absence of significant heterogeneity. The consistency and reliability of the results were further supported by the symmetrical funnel plots (**Supplementary Figure S1**) and the leave-one-out method (**Figure 3**). Notably, the remaining four components did not exhibit statistically significant associations with TPOAb-positivity (**Table 2**).

### 3.2 The causal effect of TPOAb-positive on MetS and its components

In the reverse MR Analysis, after excluding the unavailable SNPs and the palindromic SNPs, and the abnormal SNPs after RadialMR screening, we finally included 7, 8, 7, 7, 6 and 7 IVs as genetic instruments for Mets, FBG, WC, hypertension, triglycerides and HDL-C in MR analyses, respectively (**Table 3**). We had high statistical power (All SNPs have F values greater than 10) to assess associations of TPOAb-positive and MetS and its components (**Supplement Table 2**).

**Table 3:** Genetic predicted TPOAb-positivity on risk of MetS and its components in the MR analysis.

Exposure	Outcome	Method	Nsnp	Pval	OR (95%CI)	Cochran Q test P-value	P-Egger_intercept
TPOAb-positivity	Metabolic syndrome	MR Egger	7	0.852	1.011(0.907-1.127)	0.378	0.824
		Weighted median		0.998	1.000(0.959-1.043)		
		IVW		0.923	0.998(0.968-1.030)	0.496	
		Simple mode		0.803	0.991(0.933-1.054)		
		Weighted mode		0.370	1.029(0.965-1.097)		
TPOAb-positivity	FBG	MR Egger	8	0.603	0.986(0.937-1.037)	0.543	0.770
		Weighted median		0.291	0.989(0.970-1.009)		
		IVW		0.365	0.993(0.979-1.008)	0.648	
		Simple mode		0.323	0.983(0.953-1.014)		
		Weighted mode		0.303	0.982(0.950-1.014)		
TPOAb-positivity	WC	MR Egger	7	0.932	0.998(0.962-1.036)	0.326	0.801
		Weighted median		0.093	0.990(0.978-1.002)		
		IVW		0.152	0.994(0.985-1.002)	0.436	
		Simple mode		0.276	0.988(0.969-1.008)		
		Weighted mode		0.252	0.988(0.969-1.007)		
TPOAb-positivity	Hypertension	MR Egger	7	0.710	1.002(0.991-1.013)	0.995	0.991
		Weighted median		0.246	1.002(0.998-1.006)		
		IVW		0.196	1.002(0.999-1.005)	0.999	
		Simple mode		0.397	1.002(0.997-1.008)		
		Weighted mode		0.412	1.002(0.997-1.008)		
TPOAb-positivity	Triglycerides	MR Egger	6	0.530	1.014(0.974-1.055)	0.273	0.525
		Weighted median		0.712	0.997(0.983-1.011)		
		IVW		0.942	1.000(0.990-1.011)	0.330	
		Simple mode		0.752	0.996(0.975-1.018)		
		Weighted mode		0.725	0.996(0.972-1.019)		
TPOAb-positivity	HDL-C	MR Egger	7	0.720	0.993(0.959-1.029)	0.257	0.940
		Weighted median		0.556	0.996(0.984-1.009)		
		IVW		0.251	0.995(0.985-1.004)	0.364	
		Simple mode		0.613	0.995(0.976-1.014)		
		Weighted mode		0.583	0.995(0.978-1.012)		

IVW inverse-variance weighted, MetS metabolic syndrome, FBG fasting blood glucose, WC waist circumference, HDL-C high-density lipoprotein cholesterol.

As indicated in **Table 3** and the scatter plots (**Supplementary Figure S2**), the results of the MR analysis consistently indicated that neither MetS nor its five constituent elements exhibited a causal relationship with TPOAb-positivity, as reflected by odds ratios (ORs) close to 1. Furthermore, both Cochran’s Q test and Egger’s test (**Table 3**) suggested the absence of heterogeneity and potential horizontal pleiotropy in this study. The stability of the findings was further affirmed by the LOO analysis (**Supplementary Figure S3**) and funnel plots (**Supplementary Figure S4**).

## 4 Discussion

In the context of this bidirectional two-sample MR study, our findings revealed a significant and negative association between genetically predicted MetS and triglycerides with the risk of TPOAb-positivity. Conversely, the reverse MR analyses yielded no evidence suggesting that the genetic predisposition to TPOAb-positivity was linked to MetS and its components.

There are limited studies looking at the effects of MetS on TPOAb-positive. A cross-sectional study of Portuguese adults found that MetS was negatively associated with TPOAb-positive, consistent with our findings (19). Moreover, A large prospective cohort study from Iran showed that MetS subjects were less frequently TPOAb-positive than non-MetS subjects during a 10-year follow-up period (35). However, another cross-sectional study from South Korea linked TPOAb-positive to an increased incidence of MetS, in addition to abdominal obesity, low HDL-C, and elevated blood pressure (17). Different studies have come to different conclusions, which may be due to confounding factors in observational studies. Our research, which relied on genetic data sourced from extensive consortia, has unveiled a potential inverse association between Mets and TPOAb-positivity. To substantiate these findings, further investigations are imperative, involving cohort studies characterized by substantial sample sizes and prolonged follow-up periods, along with MR studies conducted across diverse populations.

As for MetS components, triglycerides were negatively associated with TPOAb-positive in this study. Several studies have shown that thyroid autoimmunity is associated with dyslipidemia, but studies have come to slightly different conclusions. A Portuguese study found a significant negative correlation between TPOAb-positivity and triglycerides composition, which is consistent with our conclusions in this study (19). However, in a Turkish study, they found a positive correlation between TPOAb-positivity and triglycerides (36). In another study, there was no significant difference in triglycerides levels between TPOAb-positive and TPOAb-negative people (37). This inconsistent result may be related to differences in factors such as race, gender, lifestyle, and age composition.

The exact mechanism of the association between triglycerides and TPOAb-positivity is unclear, but different hypotheses have been proposed. Firstly, Ghrelin is a 28-amino acid acylated peptide, serving as an effective inducer of food intake and playing a crucial role in obesity, inflammation, and autoimmune processes (38). According to reports, ghrelin can increase the synthesis of triglycerides by promoting the expression of genes related to fat production in liver cells (39). Furthermore, clinical studies indicate that thyroid function-deficient patients with high TPOAb titers exhibit decreased levels of Ghrelin (40). Thus, we believe that TPOAb-positivity seems to have a potential negative correlation with triglyceride levels. Secondly, a role for both IFN-γ and IL-4 in some murine models of experimental autoimmune Graves’ disease (EAGD) has been proposed (41). In addition, there are many studies on the pathogenesis of Type-1 helper (Th1) immune response involved in thyroid autoimmunity (42, 43). Th1 lymphocytes are recruited by Th1 chemokines secreted by damaged cells. In inflame tissue, attracted Th1 lymphocytes induce IFN-γ and TNF-α release, which stimulates the secretion of Th1 chemokines (CXCL9, CXCL10, and CXCL11), initiating and repeating the amplification feedback loop (44). These chemokines play an important role in the pathogenesis of thyroid autoimmune diseases, and studies have shown that IFN-γ induces the secretion and expression of CXCL10, CXCL9 and CXCL11 in a dose-dependent manner (45). In another study, blood sugar and triglyceride levels in mice were significantly higher when serum IFN-γ concentrations were reduced compared to controls (46). Therefore, changes in triglycerides and the associated alterations in inflammatory factors may impact the progression of autoimmune thyroid diseases. However, this is only a prediction based on existing research. It is well known that triglyceride levels are associated with obesity, and studies have reported that positive TPOAb antibodies are associated with BMI and abdominal obesity (47, 48). We know that plasma leptin levels are positively correlated with BMI and body fat. Leptin-induced inflammation and the presence of inflammatory cytokines like TNF-α and IL-6 within the thyroid gland may contribute to the development of thyroid autoimmunity (49). So triglycerides may also promote inflammation. This is a controversial topic that may require further research in the future.

The remaining MetS components including waist circumference, hypertension, HDL-C, and fasting blood glucose were not associated with TPOAb-positivity in this study. However, epidemiological studies have been inconsistent. Waist circumference is one of the indicators of obesity, and in many studies, obesity is associated with TPOAb-positive (18, 48). Gender may have played a role in the findings. A study encompassing 2,253 participants unveiled a positive correlation between waist circumference and TPOAb-positivity in men, while this association was not observed in women. In the case of women, hip circumference displayed a positive correlation with TPOAb-positivity (50). However, a nine-year follow-up study in Iran did not find an association between waist circumference and TPOAb-positivity (51). For hypertension, a study of 55,891 people in China showed significantly higher TPOAb-positive systolic and diastolic blood pressure compared to TPOAb-negative group (48). But there are also reports that there is no significant difference between the two (47). From the current published literature, the relationship between HDL-C and TPOAb-positivity may be positive, negative, or unrelated, which is also a controversial issue (37, 52, 53). This study did not find an association between fasting blood glucose and TPOAb-positivity, and a previous study has also shown no association between diabetes and TPOAb (47). However, many studies have also shown a higher prevalence of AITD in people with type 2 diabetes, so further research is needed to investigate this relationship (54, 55).

Previous studies have been controversial about the relationship between MetS and thyroid autoimmunity. We harnessed MR analysis to establish causal inferences in the bidirectional link between MetS and TPOAb-positivity. Our research boasts several strengths. Firstly, our findings successfully circumvented reverse causality and minimized the impact of residual confounding. Secondly, we capitalized on the most comprehensive summary-level data from GWAS concerning MetS and its constituent components, and TPOAb-positivity, rendering our conclusions exceptionally robust. Lastly, a battery of sensitivity analyses further enhances the reliability of our conclusions.

The study also had some limitations. The utilization of GWAS summary-level data restricted our ability to explore potential stratification effects by variables such as gender, age, lifestyle habits, or other factors. Furthermore, the TPOAb-positivity GWAS data in this study comprised summaries from various studies, each with distinct definitions of TPOAb-positivity. Therefore, it is imperative to establish a larger-scale, standardized GWAS dataset for more comprehensive analysis. In addition, some patients with chronic thyroiditis lack evidence of TPOAb-positivity. The diagnosis of thyroiditis is based on thyroid ultrasound assessment, and this subgroup of patients may not fall within the scope of the study. Lastly, the study participants exclusively comprised European residents, thus the generalizability of the findings is constrained to individuals of European descent.

## 5 Conclusion

In summary, our bidirectional MR study has unveiled a causal connection between MetS and its components and TPOAb-positivity, and no causal associations were identified in the reverse direction. Our findings imply that the SNPs of MetS and triglycerides are associated with TPOAb-positivity. However, this association would require further large clinical trials and basic studies to demonstrate a protective effect of MetS and triglycerides against thyroid autoimmunity.

## Data availability statement

Publicly available datasets were analyzed in this study. This data can be found here: https://gwas.mrcieu.ac.uk/, https://transfer.sysepi.medizin.uni-greifswald.de/thyroidomics/datasets/.

## Ethics statement

The data used in our MR analysis is entirely from previously reported summary data. Therefore, neither patient consent nor ethical approval was necessary for the study.

## Author contributions

KC: Writing – original draft, Data curation, Conceptualization. WS: Writing – review & editing, Conceptualization. LH: Writing – original draft, Funding acquisition. WD: Writing – original draft, Formal analysis. DZ: Writing – review & editing, Funding acquisition. TZ: Writing – review & editing, Funding acquisition. HZ: Writing – review & editing, Funding acquisition, Formal analysis.
