# Supplementary material for: Exploring the bidirectional relationship between metabolic syndrome and thyroid autoimmunity: a Mendelian randomization study
Source: Front Endocrinol (Lausanne). 2024 Mar 19;15:1325417. doi: 10.3389/fendo.2024.1325417 (PMC10985172; doi:10.3389/fendo.2024.1325417)
Supplement: Supplementary file 2 [file DataSheet_1.pdf]

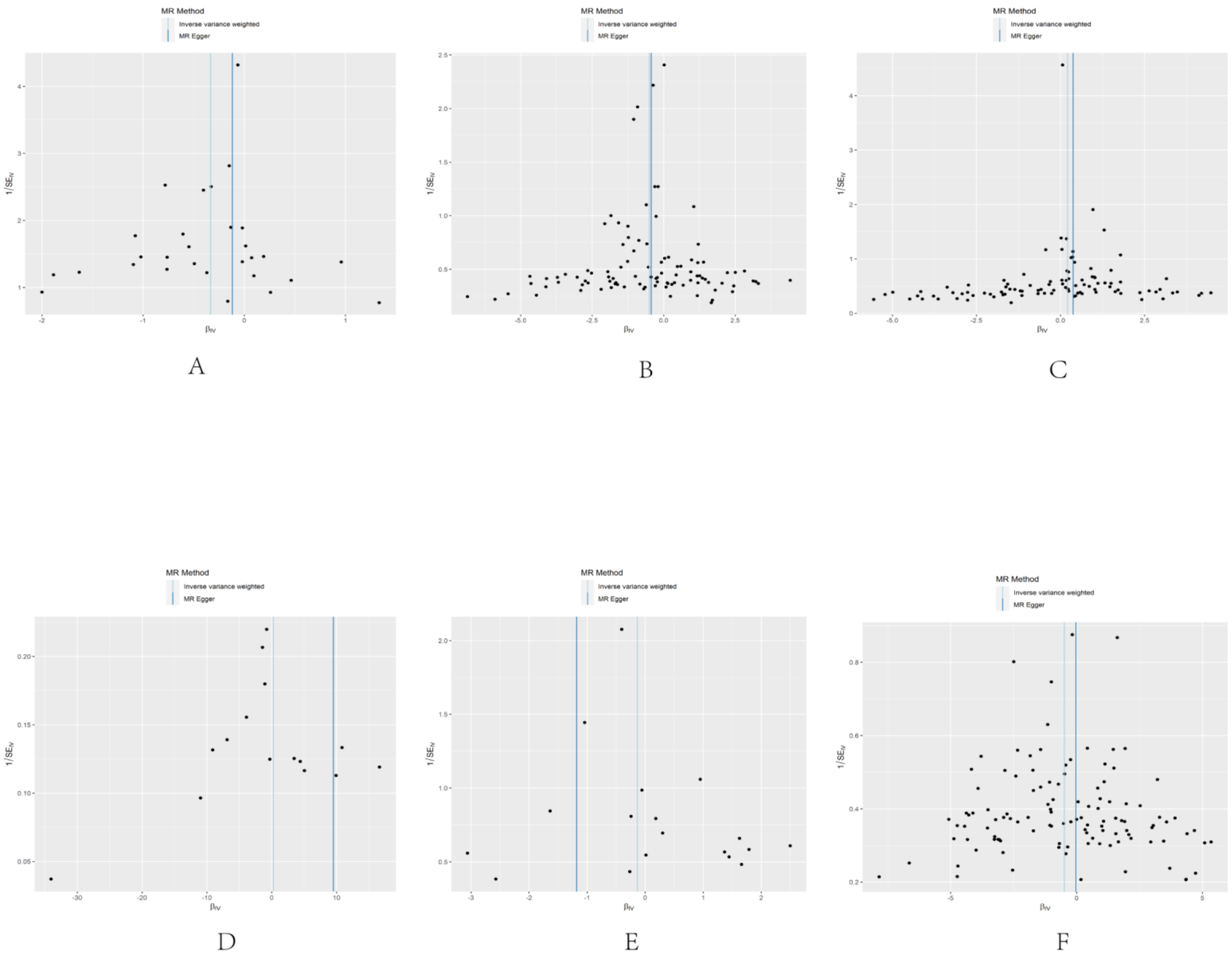

Supplementary Figure S1: The funnel plots of the association between genetic predicted MetS and its components on TPOAb-positivity in MR analysis. (A)MetS on TPOAb-positivity; (B) triglycerides on TPOAb-positivity; (C) HDL-C on TPOAb-positivity; (D) hypertension on TPOAb-positivity; (E) FBG on TPOAb-positivity; (F) WC on TPOAb-positivity. MetS, metabolic syndrome; HDL-C, high-density lipoprotein cholesterol; FBG, fasting blood glucose; WC, waist circumference.

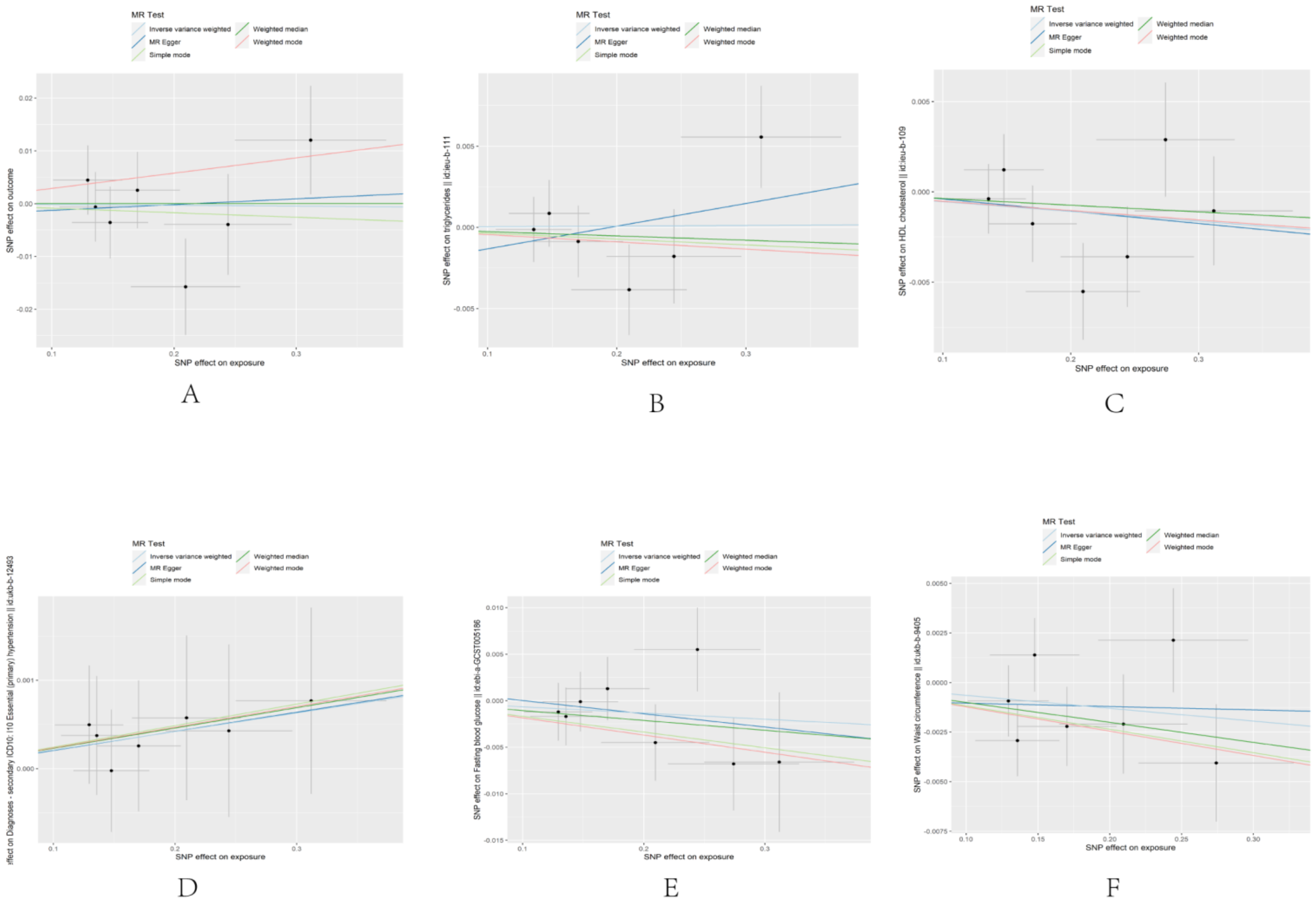

Supplementary Figure S2: The scatter plots of the association between genetically predicted TPOAb-positivity on MetS and its components. (A) TPOAb-positivity on MetS; (B) TPOAb-positivity on triglycerides; (C) TPOAb-positivity on HDL-C; (D) TPOAb-positivity on hypertension; (E) TPOAb-positivity on FBG; (F) TPOAb-positivity on WC. MetS, metabolic syndrome; HDL-C, high-density lipoprotein cholesterol; FBG, fasting blood glucose; WC, waist circumference.

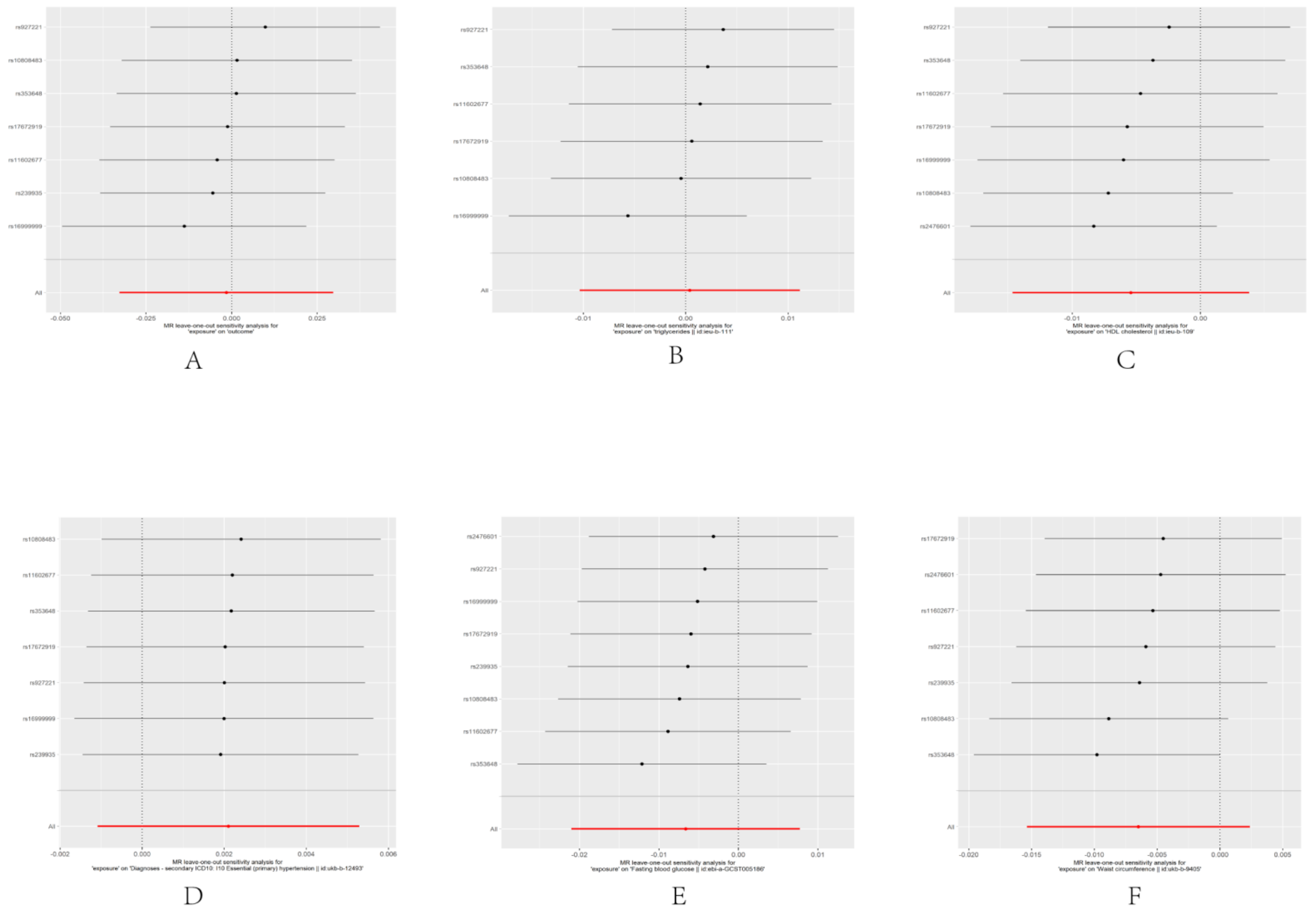

Supplementary Figure S3: The leave-one-out analysis of the association between genetically predicted TPOAb-positivity on MetS and its components. (A) TPOAb-positivity on MetS; (B) TPOAb-positivity on triglycerides; (C) TPOAb-positivity on HDL-C; (D) TPOAb-positivity on hypertension; (E) TPOAb-positivity on FBG; (F) TPOAb-positivity on WC. MetS, metabolic syndrome; HDL-C, high-density lipoprotein cholesterol; FBG, fasting blood glucose; WC, waist circumference.

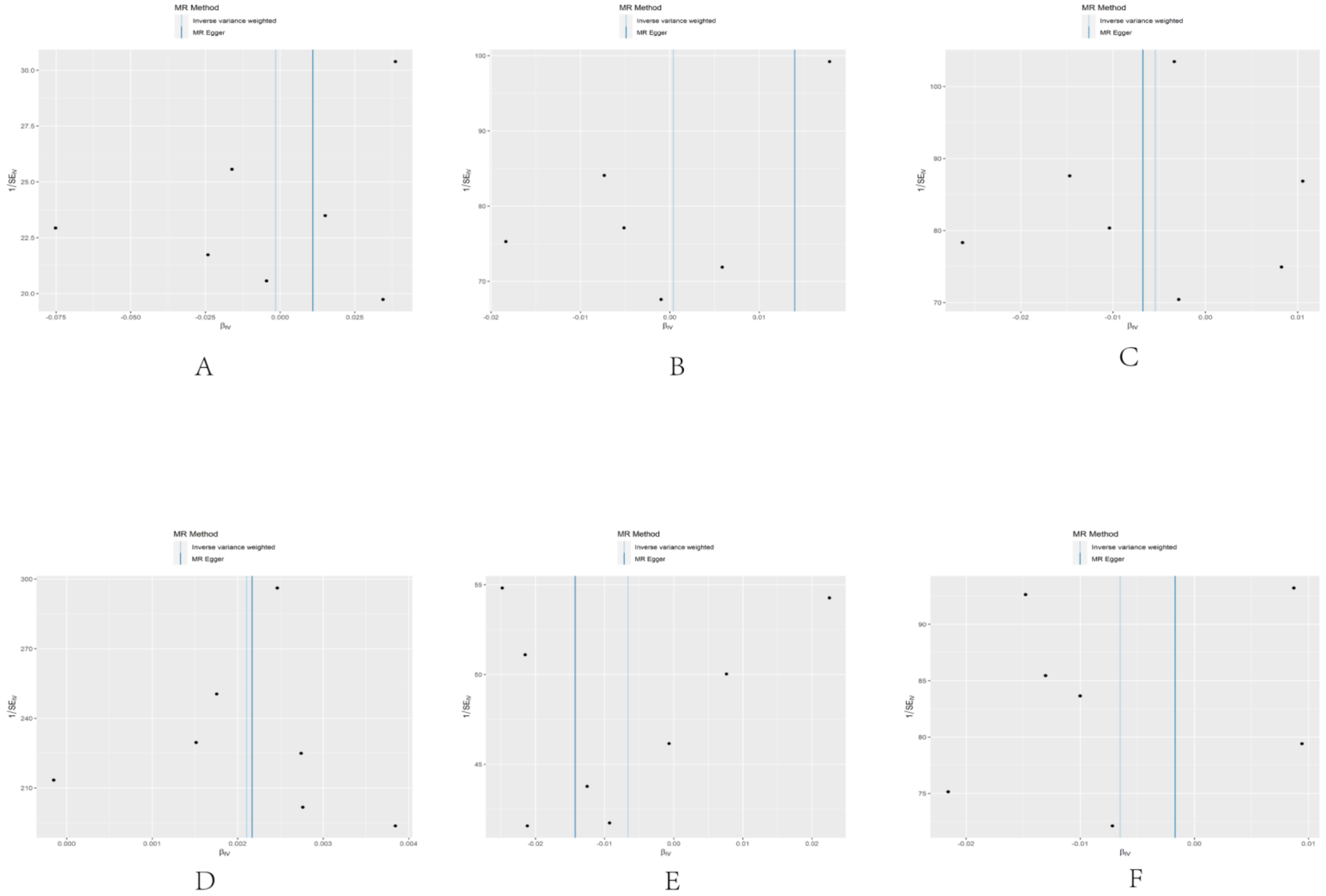

Supplementary Figure S4: The funnel plots of the association between genetically predicted TPOAb-positivity on MetS and its components. (A) TPOAb-positivity on MetS; (B) TPOAb-positivity on triglycerides; (C) TPOAb-positivity on HDL-C; (D) TPOAb-positivity on hypertension; (E) TPOAb-positivity on FBG; (F) TPOAb-positivity on WC. MetS, metabolic syndrome; HDL-C, high-density lipoprotein cholesterol; FBG, fasting blood glucose; WC, waist circumference.
